# A randomized controlled study evaluating the head-lift exercise in head and neck cancer patients with radiation-induced dysphagia: effect on swallowing function and health-related quality of life over 12 months

**DOI:** 10.1007/s00405-023-08183-7

**Published:** 2023-08-16

**Authors:** Kerstin Petersson, Caterina Finizia, Nina Pauli, Hans Dotevall, Lisa Tuomi

**Affiliations:** 1 Department of Otorhinolaryngology, Head and Neck Surgery, Institute of Clinical Sciences, Sahlgrenska Academy, University of Gothenburg, Gothenburg, Sweden; 2 Department of Otorhinolaryngology, Head and Neck Surgery, Sahlgrenska University Hospital, Region Västra Götaland, 41345 Gothenburg, Sweden; 3 Institute of Neuroscience and Physiology, Speech and Language Pathology Unit, Sahlgrenska Academy, University of Gothenburg, Gothenburg, Sweden

**Keywords:** Head-lift exercise, Head and neck cancer, Dysphagia, Health-related quality of life, Radiotherapy, Randomized controlled trial

## Abstract

**Purpose:**

Dysphagia is common after radiotherapy for head and neck cancer (HNC) and can affect health-related quality of life (HRQL). This randomized controlled trial aimed to evaluate the effect of the head-lift exercise (HLE) over 12 months in HNC patients with radiation-induced dysphagia.

**Methods:**

Sixty-one patients with dysphagia were randomized to intervention group (*n* = 30) and control group (*n* = 31) at 6–36 months after completion of radiotherapy for HNC. Dysphagia-specific HRQL was measured with the MD Anderson Dysphagia Inventory (MDADI); general and HNC-specific HRQL was measured with the European Organization for Research and Treatment of Cancer Quality of Life questionnaire Core 30 (EORTC QLQ-C30) and QLQ-H&N35. Measurements were made at baseline, and at 8 weeks and 12 months after start of intervention.

**Results:**

Adherence to the intervention was good throughout the year. When comparing change from baseline reports to each follow-up no statistically significant differences between the groups were found in any of the HRQL instruments. There were some statistically significant changes within groups compared to baseline. The intervention group improved self-rated swallowing function on the MDADI at 8 weeks (emotional domain, *p* = 0.03; functional domain, *p* = 0.007; total score, *p* = 0.01) and the control at twelve months (emotional domain, *p* = 0.03; functional domain, *p* = 0.02; physical domain, *p* = 0.004; total score, *p* = 0.002).

**Conclusion:**

In this randomized control study, no effect was observed short term or at 12 months on HRQL after use of the HLE as rehabilitation for radiation-induced dysphagia.

## Introduction

Head and neck cancer (HNC) incidence is expected to increase in the western world mainly as a consequence of an aging population and an increase of HNC caused by human papillomavirus (HPV) [1]. Around 50% of HNC patients are afflicted by dysphagia, i.e., swallowing difficulties [2, 3].

Radiotherapy with or without chemotherapy is an effective oncological treatment, offered in some extent to around 80% of HNC patients [4]. Dysphagia can arise as an acute side effect due to mucositis, pain, and edema, and subside sometime after treatment, or as a permanent side effect caused by fibrosis or cranial neuropathy in the irradiated area [5].

Dysphagia impacts several aspects of HNC patients’ lives and is known to have negative effects on the patients’ health-related quality of life (HRQL) [1]. Patients report that swallowing difficulties leave them feeling tired and weak, thereby affecting their general activity levels [6]. Additionally, embarrassment and anxiety over swallowing difficulties result in lack of pleasure-eating, of going out to eat, and of eating in the company of family and friends, which in turn brings feelings of isolation and loss of personal self [6, 7].

Dysphagia treatment aims to prevent or decrease medical risks of dysphagia such as malnutrition, dehydration, aspiration pneumonia, and choking and to improve HRQL [1]. Intervention often includes modification of food and drink to consistencies that are less prone to enter the airway and that leave less pharyngeal residue [1]. Modification only improves swallowing function temporarily and can have negative consequences as it is associated with dehydration, weight loss, and decreased HRQL [8]. Over time, focus on specific swallowing exercises that intend to improve function by increasing range of motion and strengthening of muscles in the oral and pharyngeal area has grown [1]. However, there is a lack of conclusive evidence on the effectiveness of swallowing exercises for HNC patients, mainly due to methodological shortcomings such as small research samples, several interventions included in exercise protocols, as well as lack of reports on adherence to exercises and on results from subsequent follow-up periods [1, 9, 10]. Furthermore, there are few studies containing a control group that evaluate the effect of intervention on HRQL [9, 10]. As most patients prefer not to modify their diet [1], efficient interventions that improve swallowing function as well as HRQL are of importance.

The head-lift exercise (HLE) was originally developed to improve the upper esophageal sphincter (UES) by strengthening the suprahyoidal, thyrohyoid, and pharyngeal muscles to improve hyoid and laryngeal elevation and consequently the UES opening [11]. The HLE has been used as an intervention for dysphagia among HNC patients for several years [12] as research studies have showed some evidence of less aspiration during swallowing and less post-swallow residue as well as better preservation of the UES opening, hyoid bone movement, and strengthened suprahyoid muscles [13–15]. However, results from a large, randomized control trial have previously been lacking.

The research group has recently published two articles from a randomized control trial evaluating the effect of the HLE on swallowing function and physiology in HNC by observer-rated instrumental assessment [16, 17]. In these studies, no apparent support for previous data of improved swallowing function and physiology were found. However, some positive effects on patient-reported swallowing were seen after 8 weeks of intervention. Effect of the HLE has not yet been evaluated at 12 months in the present cohort and there is a lack of studies evaluating effect of swallowing intervention from subsequent follow-ups within the research area [10]. The aim of this randomized study was to evaluate the effect over 12 months of HLE treatment in HNC patients with radiation-induced dysphagia. The evaluation was focused on patients’ perception of general and dysphagia-specific HRQL over a period of 12 months.

## Materials and methods

### Subjects

**Fig. 1:**
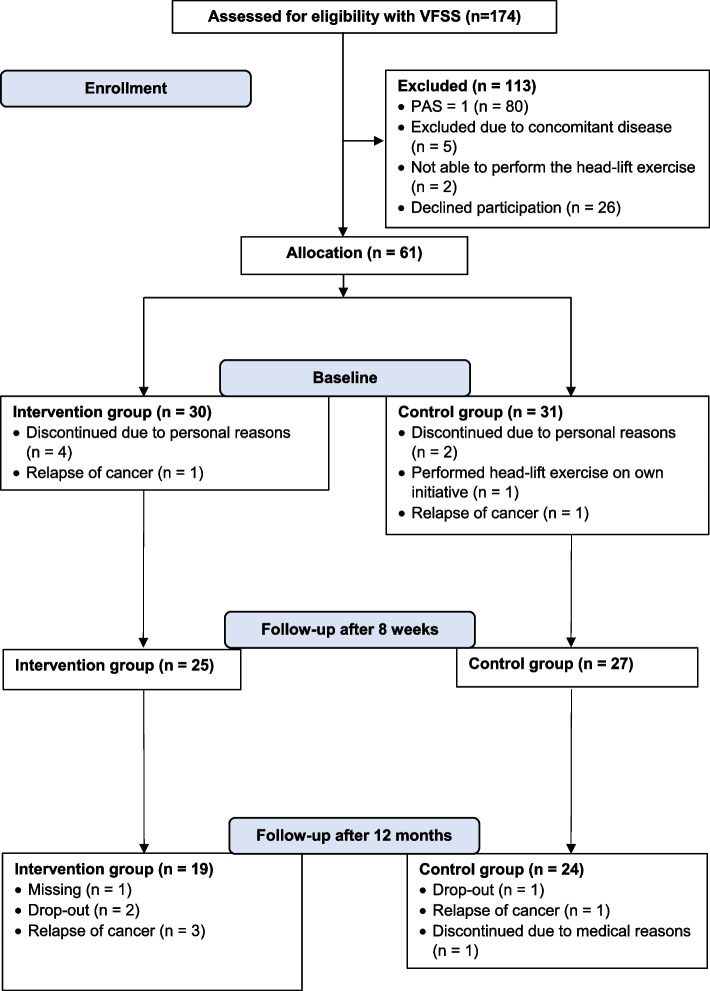
Flow chart of enrollment

Adult patients with HNC treated at Sahlgrenska University Hospital, Gothenburg, Sweden were eligible for the study. Inclusion criteria were: tumor of the base of the tongue, tonsil, hypopharynx, or larynx 6–36 months after completion of radiotherapy. Eligible patients were contacted by telephone and offered a videofluoroscopic examination of swallowing (VFSS) to determine presence of swallowing difficulties. 174 patients accepted (see Fig. 1). Patients who presented with at least PAS 2 according to the penetration–aspiration scale (PAS) [18] on two swallows during VFSS (*n* = 87) were invited to participation. Exclusion criteria were laryngectomy, tracheostomy, surgical treatment for HNC (other than tonsillectomy for diagnostic purposes), previous treatment for HNC, neurological or neuromuscular disease, previous swallowing difficulties not connected to current HNC diagnosis, inability to swallow any bolus, or inability to perform the HLE.

### Oncologic treatment

All patients received treatment in accordance with the regional cancer treatment program. External Beam Radiation Therapy (EBRT) was typically delivered by intensity modulated/volumetric modulated radiation therapy (IMRT/VMAT). Radiotherapy was either delivered once daily (conventional, *n* = 47) or twice daily (accelerated, *n* = 5). Most patients received radiation to a total of 68 Gy with 2 Gy fractions once daily, 5 days per week. Chemotherapy was typically 5–6 cycles of cisplatin given either as concomitant (*n* = 33) or as induction (*n* = 9) to radiotherapy. Ten patients received no chemotherapy. Seventeen patients received brachytherapy.

### Study design

Patients who accepted participation in the study (*n* = 61) were included and randomized through optimal allocation according to Pocock's sequential randomization method based on: PAS score at time of inclusion, tumor location, tumor stage, age, gender, and comorbidity according to the Adult Comorbidity Evaluation-27 index (ACE-27) [19]. However, as six patients chose to discontinue due to personal reasons, two had relapse of cancer, and one practiced HLE on their own initiative, only 52 patients (25 in the intervention group and 27 in control group) are presented in this study (see Fig. 1). HRQL was measured at each study occasion: baseline, 8 weeks, and 12 months after the first follow-up.

Patients in the intervention group performed HLE and received standard dysphagia care offered at the clinic while the control group only received standard dysphagia care. Standard care included advice about food and drink as well as the use of head positioning and swallowing maneuvers (the supraglottic swallow, effortful swallow, Mendelsohn maneuver) during meals and was based on the VFSS and flexible endoscopic evaluation of swallowing (FEES).

### HLE intervention

Patients in the intervention group were instructed by a study speech and language pathologist (SLP) and received written and video instructions and a training diary. One session of HLE consisted of three head lifts in supine position sustained for 60 s and with a 60-s rest in between and 30 consecutive repetitions of head lifts. During the first 8 weeks, patients performed three HLE sessions per day. The first 2 weeks, the study SLP met with the patient two times per week to monitor training technique. The remainder of the period, technique was supervised once every 2 weeks with follow-ups by telephone in between meetings. Only the intervention group had contact with the study SLP during the initial period. After 8 weeks up to 12 months post-intervention, the intervention group was encouraged to continue to do one HLE session per day or a minimum of three sessions per week.

There were no statistically significant differences between the groups regarding the given standard care (previously published [16]).

### Assessment and endpoints

#### VFSS

VFSS was performed to assess eligibility for inclusion by a gastrointestinal radiologist in collaboration with an experienced study SLP. During VFSS, patients were offered barium contrast in varying consistencies and different amounts. VFSS method is detailed elsewhere [16].

##### The MD Anderson Dysphagia Inventory

The MD Anderson Dysphagia Inventory (MDADI) was developed to measure dysphagia-specific HRQL in HNC patients [20] and is frequently used both in clinical and research contexts [21, 22]. It has been translated and validated into a Swedish version with retained psychometric properties [23]. The Swedish MDADI version has been found to be sensitive to change over time [24]. The instrument contains 20 items divided into four domains. The emotional domain (six items) reflects how the patients’ feelings are affected. The functional domain (five items) measures effect on daily life, while the physical domain (eight items) represents the patient’s own perception of swallowing difficulties. The global domain consists of one item concerning overall impact of swallowing function on everyday activities. Patients respond using a Likert scale from one to five (strongly agree to strongly disagree). Domain scores range from 20 to 100 and higher scores represent better functioning. Based on anchor-based analysis a 10-point difference in total score between groups has been suggested as a clinically relevant difference [25]. The same study calculated distribution based minimal clinically important differences (MCID) between groups for domain scores and showed that 8.6 (emotional), 9.2 (functional), and 9.3 (physical) could be considered a clinically relevant difference. As a cut-off for moderate–severe swallowing disability, a total score less than 60 has been used in previous studies [24, 26].

##### European Organization for Research and Treatment of Cancer Quality of Life Questionnaire

The European Organization for Research and Treatment of Cancer Quality of Life Questionnaire Core 30 (EORTC QLQ-C30) is a cancer-specific HRQL instrument and consists of 30 items. Two items reflect overall global health status. The remaining items are divided into five functional and nine symptom scales.

The EORTC head and neck cancer module (QLQ-H&N35) is designed to be used in combination with the EORTC QLQ-C30 and focuses on issues specific for the HNC population. It consists of 35 items divided into seven symptoms scales and eleven single items.

Items are responded to using Likert scales and responses are converted into scores from 0 to 100. Higher scores reflect better HRQL on the functional scales and higher levels of symptoms on the symptoms scales [27]. A change of 10 p or more is considered a MCID on the EORTC QLQ-C30 [28].Only domains and items that were hypothesized to be affected by the HLE are presented in this study.

All of the above-mentioned HRQL instruments are considered reliable, valid, and are used both in clinical and research settings.

### Statistical analysis

End points concerning HRQL presented in this study are exploratory as power calculations were based on PAS. Sample size to achieve a predicted power of 80% for a one-step change on the PAS with a standard deviation of 1.2 was calculated to 30 participants in each group including potential dropouts. Calculations were made using the Mann–Whitney *U* test with a significance level of *p* < 0.05.

All statistical analyses were performed using SAS version 9.4. For descriptive purposes, results are presented as mean, standard deviation, and 95% confidence intervals (CI) for continuous variables and as numbers and percentages for categorical variables. All tests performed were two-tailed non-parametric tests, with a significance level of *p* < 0.05 throughout.

The Fisher’s non-parametric permutation test was used to calculate mean differences between groups for continuous variables. Comparisons of differences within each group were made using the Fisher’s non-parametric permutation test for paired observations for continuous variables. Ordered categorical variables were calculated using the Mantel–Haenszel test, and non-ordered categorical using the Chi-square test. For dichotomous variables, the Fisher’s exact test was used.

### Ethical considerations

The study was approved by the Regional Ethical Review Board in Gothenburg, Sweden and was conducted according to the Declaration of Helsinki. All participants gave their written informed consent before inclusion in this study. The study population have previously been described in part, using objective instrumental assessments [16, 17].

## Results

An overview of patient participation and follow-ups is presented in Fig. 1. Comparisons of social demographics and treatment data between the intervention and control group at baseline are presented in Table 1. The same variables were also compared between the groups at 12 months. No statistically significant differences between the intervention and control groups were found for sociodemographic and treatment data at either baseline or 12-month follow-up. Comparison of nutritional and swallowing function variables at baseline and 12 months revealed no statistically significant differences between the intervention and control groups (Table 2). Drop-out analysis comparing baseline data from the participants who dropped out during the study year (*n* = 9) to the participants remaining at 12 months (*n* = 43) revealed that no patient among the dropouts had received brachytherapy. Additionally, among the dropouts, there was a higher proportion of hypopharyngeal tumors (2/9 vs 2/43).

**Table 1:** Sociodemographic and treatment data of the participants in the intervention and control group at baseline

Variables	Intervention group (*n* = 25)	Control group (*n* = 27)
	Mean (SD) Median (min; max)	Mean (SD) Median (min; max)
Age (years)	63.7 (8.4) 63 (45; 80)	63.7 (6.7) 63 (50; 75)
Time since completion of radiotherapy (months)	11.2 (5.9) 9 (6; 29)	13.0 (8.1) 9 (6; 37)

	*n* (%)^a^	*n* (%)^a^
Sex		
Male	18 (72)	21 (78)
Female	7 (28)	6 (22)
Education		
Elementary school	9 (36)	9 (33)
High school 2–4 years	11 (44)	8 (30)
College/university	5 (20)	10 (27)
Lives alone	6 (24)	10 (37)
Working	11 (44)	16 (59)
Risk use of alcohol	3 (12)	4 (15)
Smoking status		
Never smoked	7 (28)	6 (22)
Quit > 12 months ago	11 (44)	14 (52)
Quit < 12 months ago	4 (16)	3 (11)
Current smoker	3 (12)	4 (15)
Tumor location		
Tonsil	11 (44)	10 (37)
Base of tongue	10 (40)	10 (37)
Hypopharynx	1 (4)	3 (11)
Larynx	3 (12)	4 (15)
TNM stage		
I	1 (4)	2 (7)
II	4 (16)	2 (7)
III	1 (4)	2 (7)
IV	19 (76)	21 (78)
Radiotherapy	5 (80)	5 (19)
Chemoradiation therapy	20 (80)	22 (82)
Brachytherapy	10 (40)	7 (26)
Comorbidity*		
None	11 (22)	8 (30)
Mild	12 (48)	12 (44)
Moderate	1 (4)	6 (22)
Severe	1 (4)	1 (4)

There were no statistically significant differences between the groups at baseline

^a^Percentages rounded, therefore does not always sum to 100

*Graded according to the ACE-27 Adult Comorbidity Evaluation-27

**Table 2:** Descriptive characteristics of nutritional and swallowing function variables in the intervention and control group

Variable	Baseline		12 months	
	Intervention group (*n* = 25) *n* (%)^a^	Control group (*n* = 27) *n* (%)^a^	Intervention group (*n* = 19) *n* (%)^a^	Control group (*n* = 24) *n* (%)^a^
BMI classification				
Under weight (< 18.5)	4 (16)	3 (11)	1 (5)	4 (17)
Normal weight (18.5–24.9)	18 (72)	17 (63)	11 (58)	12 (50)
Over weight (> 25)	3 (12)	7 (26)	7 (37)	8 (33)
Salivary flow				
Hyposalivation (≤ 0.7 ml/min)	11 (44)	7 (26)	2 (11)	4 (17)
Missing	0 (0)	0 (0)	3 (16)	2 (8)
Mouth opening				
Trismus (≤ 35 mm)	3 (12)	5 (19)	4 (21)	5 (21)
Missing	1 (4)	0 (0)	0 (0)	1 (4)
Feeding tube				
Feeding tube use	1 (4)	4 (15)	2 (11)	1 (4)
Missing	0 (0)	0 (0)	1 (5)	1 (4)
Aspiration pneumonia				
Yes	3 (12)	1 (4)	1 (5)	0 (0)
Missing	0 (0)	0 (0)	1 (5)	1 (4)
MDADI total score < 60	7 (28)	6 (22)	4 (21)	2 (8)

There were no statistically significant differences between the groups at baseline or at the 12-month follow-up

*BMI* body mass index, *MDADI* The M.D. Anderson Dysphagia Inventory, total score < 60 represent impaired swallowing function

^a^Percentages rounded, therefore does not always sum to 100

### Adherence to training

During the first 8 weeks after baseline, patients were instructed to exercise three times per day, i.e., 21 occasions per week. The mean number of training sessions performed by the intervention group during the 8 weeks was 18.7 (89%).

After the 8-week follow-up, participants were recommended to exercise once daily, or a minimum of three sessions per week for 12 months and the mean number of performed training sessions per week was 3.0.

### MDADI

Analysis of change in dysphagia-specific HRQL from baseline to follow-up at 8 weeks and 12 months revealed no apparent effect of the HLE intervention. There were no statistically significant differences found in any of the domains nor in the total score between the intervention and control groups (Table 3). Furthermore, when analyzing between group results none of the differences reached the thresholds for clinical significance [25].

**Table 3:** Mean scores for MDADI at each follow-up for intervention and control group. Change from baseline to each follow-up within and between groups

	Intervention					Control					Difference (Δ) in change between groups from baseline	
	Baseline	8 weeks	12 months	Δ Baseline-8 weeks	Δ Baseline-12months	Baseline	8 weeks	12 months	Δ Baseline-8 weeks	Δ Baseline-12 months	Δ Baseline-8 weeks	Δ Baseline-12 months
	Mean (SD)	Mean (SD)	Mean (SD)	Mean (95% CI)	Mean (95% CI)	Mean (SD)	Mean (SD)	Mean (SD)	Mean (95% CI)	Mean (95% CI)	Mean (95% CI)	Mean (95% CI)
Emotional	81.4 (19.3)	89.1 (16.8)	86.6 (22.3)	6.0 (0.8; 11.3)*	2.9 (− 3.0; 8.7)	86.2 (16.2)	88.4 (16.6)	92.4 (12.2)	2.3 (− 0.9; 5.4)	5.8 (0.7; 10.9)*	3.7 (− − 2.1; 9.5)	− 2.9 (− 10.4; 4.6)
Functional	77.1 (21.4)	87.2 (23.1)	85.3 (22.6)	10.0 (3.2; 17.1)**	4.4 (− 0.9; 9.8)	80.9 (24.4)	85.2 (18.5)	89.8 (19.2)	4.30 (0.0; 8.7)	8.0 (1.2; 15.4)*	5.7 (− 2.2; 13.7)	− 3.6 (− 13.0; 5.2)
Physical	69.6 (19.6)	76.1 (18.8)	75.4 (20.0)	6.5 (− 0.1; 13.3)	2.8 (− 2.3; 8.1)	67.7 (20.8)	74.6 (18.2)	79.8 (19.3)	6.9 (2.4; 11.3)**	10.0 (3.6; 16.3)**	− 0.4 (− 8.1; 7.3)	− 7.1 (− 15.4; 1.1)
Global	77.5 (29.7)	87.5 (24.9)	83.2 (24.3)	− 6.7 (− 16.7; 2.9)	1.1 (− 7.5; 10.0)	70.4 (29.0)	75.6 (30)	81.7 (22.8)	5.2 (− 5.0; 15.4)	7.5 (− 2.2; 17.1)	1.8 (− 12.9; 16.7)	− 6.4 (− 20.0; 7.3)
Total	71.4 (17.9)	79.8 (16.3)	77.4 (19.1)	7.0 (1.7; 12.5)**	3.1 (− 0.8; 7.1)	73.2 (17.7)	77.7 (15.7)	82.2 (14.9)	4.5 (1.7; 7.2)**	7.7 (2.9; 12.6)**	2.6 (− 3.3; 8.3)	− 4.6 (− 11.0; 1.5)

Higher scores represent better functioning

*MDADI* The M.D. Anderson Dysphagia Inventory

*p ≤ 0.05, **p ≤ 0.01, ***p ≤ 0.001

Some changes occurred within groups over time (Table 3). The intervention group demonstrated improvements from baseline results after the most intensive training period, i.e., to the follow-up at 8 weeks, with statistically significant improvements in the emotional and functional domain as well as in total score (*p* = 0.03, *p* = 0.007, *p* = 0.01, respectively). This improvement decreased and was no longer statistically significant in comparison to baseline results at the 12-month follow-up. The control group demonstrated several improvements when comparing baseline results to the 12-month follow-up, with statistically significant differences in the emotional, functional, and physical domains and in total score (*p* = 0.03, *p* = 0.02, *p* = 0.004, *p* = 0.002, respectively).

### EORTC QLQ-C30

There were no statistically significant differences between groups concerning change in cancer-related HRQL from baseline to follow-up at 8 weeks and 12 months (Table 4). Some statistically significant improvements were seen over time in the intervention group, in the role, social function and global quality of life domains (*p* = 0.05, *p* = 0.01, *p* = 0.02, respectively).

**Table 4:** Mean scores for EORTC QLQ-C30 at each follow-up for intervention and control group. Change from baseline to each follow-up within and between groups

	Intervention					Control					Difference (Δ) in change between groups from baseline	
	Baseline	8 weeks	12 m	Δ Baseline-8 weeks	Δ Baseline-12 months	Baseline	8 weeks	12 months	Δ Baseline-8 weeks	Δ Baseline-12 months	Δ Baseline-8 weeks	Δ Baseline-12 months
	Mean (SD)	Mean (SD)	Mean (SD)	Mean (95% CI)	Mean (95% CI)	Mean (SD)	Mean (SD)	Mean (SD)	Mean (95% CI)	Mean (95% CI)	Mean (95% CI)	Mean (95% CI)
Physical	87.2 (15.9)	90.1 (14.7)	89.1 (15.9)	2.9 (0.0; 5.6)	1.4 (− 2.2; 5.0)	88.1 (11.3)	89.6 (11.0)	90.8 (9.8)	1.5 (− 2.4; 5.3)	2.2 (− 1.7; 6.0)	1.5 (− 3.3; 6.2)	− 0.8 (− 6.1; 4.7)
Role	80.0 (27.6)	87.3 (26.0)	86.8 (22.6)	7.3 (1.1; 14.1)*	5.3 (− 1.7; 12.5)	78.4 (27.7)	82.7 (24.7)	79.2 (27.9)	4.3 (− 2.1; 10.6)	0.0 (− 14.6; 13.9)	3.0 (− 6.1; 12.2)	5.3 (− 11.7; 22.2)
Emotional	84.4 (20.0)	85.7 (20.5)	86.0 (19.8)	1.2 (− 2.5; 4.9)	0.0 (− 4.2; 4.2)	85.2 (17.2)	88.3 (14.7)	88.9 (12.7)	3.1 (− 3.9; 10.0)	5.6 (− 0.8; 12.0)	− 1.9 (− 9.6; 6.0)	− 5.6 (− 13.6; 2.5)
Cognitive	87.3 (15.4)	86.7 (16.0)	81.6 (22.1)	− 0.7 (− 6.7; 5.6)	− 7.9 (− 16.7; 1.9)	88.3 (12.9)	82.7 (17.0)	88.9 (11.7)	− 5.6 (− 13.0; 1.5)	2.1 (− 2.8; 6.7)	4.9 (− 4.2; 14.1)	− 10.0 (− 19.7; − 0.0)
Social	82.7 (25.2)	85.3 (25.6)	91.2 (16.1)	2.7 (− 3.6; 9.0)	6.1 (2.1; 10.0)**	86.4 (19.1)	88.9 (17.9)	88.2 (21.1)	2.5 (− 6.0; 10.6)	0.0 (− 10.0; 9.3)	0.2 (− 10.0; 10.4)	6.1 (− 4.6; 16.7)
Global quality of life	75.0 (22.0)	80.7 (19.4)	82.5 (20.4)	5.7 (− 1.7; 12.9)	7.5 (1.7; 13.5)*	72.8 (19.4)	75.6 (17.4)	78.5 (19.2)	2.8 (− 2.5; 8.3)	4.5 (− 4.2; 13.0)	2.9 (− 5.8; 11.7)	2.9 (− 7.5; 13.5)

Higher scores represent better functioning

*EORTC QLQ-C30* European Organization for Research and Treatment of Cancer Quality of Life questionnaire Core 30

*p ≤ 0.05, **p ≤ 0.01, ***p ≤ 0.001

### EORTC QLQ-H&N35

Analysis of change from baseline to each follow-up revealed no statistically significant differences between groups (Table 5). However, there was a clinically relevant difference between groups for sticky saliva where the intervention group reported less symptoms compared to the control group both at baseline and the 6 months follow-up. Similar to the results of the emotional and functional domains and the total score of the MDADI, self-rated swallowing function assessed with the EORTC QLQ-H&N35 increased from baseline to 8 weeks in the intervention group (*p* = 0.04), but not from baseline to follow-up at 12 months.

**Tabel 5:** Mean scores for EORTC QLQ-H&N35 at each follow-up for intervention and control group and change from baseline to each follow-up within and between groups

	Intervention					Control					Difference (Δ) in change between groups from baseline	
	Baseline	8 weeks	12 months	Δ Baseline-8 weeks	Δ Baseline-12 months	Baseline	8 weeks	12 months	Δ Baseline-8 weeks	Δ Baseline-12 months	Δ Baseline-8 weeks	Δ Baseline-12 months
	Mean (SD)	Mean (SD)	Mean (SD)	Mean (95% CI)	Mean (95% CI)	Mean (SD)	Mean (SD)	Mean (SD)	Mean (95% CI)	Mean (95% CI)	Mean (95% CI)	Mean (95% CI)
Pain	13.0 (13.4)	8.7 (11.9)	11.0 (11.5)	− 4.3 (− 8.3; − 0.0)	− 4.4 (− 12.0; 3.1)	21.9 (25.0)	16.4 (22.3)	12.5 (16.3)	− 5.6 (− 11.8; 0.6)	− 11.5 (− 21.5; − 1.7)*	1.22 (− 6.3; 8.9)	7.07 (− 5.3; 20.1)
Swallowing	28.7 (23.5)	21.3 (21.0)	24.6 (23.8)	− 7.3 (− 14.2; − 0.7)*	− 0.9 (− 9.4; 7.23)	29.6 (23.3)	23.8 (23.2)	17.0 (16.4)	− 5.9 (− 13.5; 1.9)	− 8.3 (− 22.2; 5.6)	− 1.5 (− 11.4; 8.9)	7.5 (− 3.8; 18.9)
Senses	25.3 (25.1)	18.7 (18.8)	27.2 (27.9)	− 6.7 (− 14.1; − 0.0)	2.6 (− 9.5; 14.8)	18.5 (22.8)	19.1 (24.3)	21.5 (27.6)	0.6 (− 4.2; 5.6)	0.7 (− 5.6; 7.4)	− 7.3 (− 15.5; 1.4)	1.9 (− 11.1; 15.0)
Social contact	8.3 (15.7)	6.9 (17.2)	5.3 (12.9)	− 1.3 (− 3.6; 1.0)	− 2.1 (− 6.7; 1.8)	4.2 (7.9)	2.5 (5.6)	1.9 (4.6)	− 1.7 (− 5.0; 1.5)	− 1.7 (− 4.2; 0.7)	0.4 (− 3.6; 4.4)	− 0.4 (− 5.3; 4.4)
Social eating	24.3 (25.0)	19.3 (26.1)	18.9 (25.3)	− 5.0 (− 11.7; 1.7)	− 1.8 (− 11.9; 8.3)	21.0 (18.7)	18.5 (23.5)	12.8 (15.3)	− 2.5 (− 7.6; 3.0)	− 6.9 (− 13.6; − 0.0)	− 2.5 (− 11.1; 5.8)	5.2 (− 6.5; 16.7)
Teeth	18.7 (32.0)	13.3 (27.2)	14.0 (30.1)	− 5.3 (− 12.1; − 0.0)	− 3.5 (− 14.3; 6.7)	14.8 (29.7)	17.3 (26.7)	20.8 (32.3)	2.5 (− 9.5; 14.6)	4.2 (− 7.4; 16.7)	− 7.8 (− 21.2; 5.6)	− 7.7 (− 23.3; 8.3)
Opening mouth	18.7 (27.4)	10.7 (23.0)	15.8 (28.0)	− 8.0 (− 16.7; − 0.0)	− 1.8 (− 18.2; 14.8)	18.5 (31.1)	16.0 (21.4)	13.9 (25.9)	− 2.5 (− 10.0; 4.8)	− 6.9 (− 12.8; − 0.0)	− 5.5 (− 16.7; 5.6)	5.2 (− 10.0; 20.8)
Dry mouth	62.7 (36.4)	54.7 (34.5)	47.4 (33.9)	− 8.0 (− 19.4; 3.3)	− 8.8 (− 18.5; − 0.0)	58.0 (34.1)	56.8 (35.6)	51.4 (38.0)	− 1.2 (− 9.1; 6.7)	− 9.7 (− 19.4; − 0.0)	− 6.8 (− 20.0; 6.7)	1.0 (− 12.1; 13.9)
Sticky saliva	42.7 (31.2)	32.0 (32.6)	40.4 (39.4)	− 10.7 (− 23.1; − 0.0)	1.8 (− 16.7; 20.0)	55.6 (32.0)	46.9 (33.7)	47.2 (31.0)	− 8.6 (− 22.2; 4.8)	− 8.3 (− 22.2; 5.6)	− 2.0 (− 20.0; 16.7)	10.1 (− 12.1; 33.3)
Felt ill	10.7 (23.0)	4.0 (11.1)	10.5 (19.4)	− 6.7 (− 13.3; − 0.00)	1.8 (− 6.7; 10.0)	11.1 (20.7)	11.1 (16.0)	12.5 (19.2)	− 0.0 (− 8.3; 8.3)	1.4 (− 6.7; 9.1)	− 6.7 (− 16.7; 3.3)	0.4 (− 11.1; 11.1)

Lower scores represent better functioning

*EORTC QLQ-H&N35* European Organization for Research and Treatment of Cancer Quality of Life questionnaire head and neck module

*p ≤ 0.05, **p ≤ 0.01, ***p ≤ 0.001

## Discussion

The purpose of this randomized controlled study was to evaluate effect of HLE on patients’ perception of general and dysphagia-specific HRQL over time in patients with radiation-induced dysphagia. However, results did not reveal any convincing improvements in patients’ perception of general and dysphagia-specific HRQL in the intervention group. The current study makes an important contribution to the research field as it is, to our knowledge, the only larger randomized controlled study evaluating effect of the HLE on patients’ perception of swallowing and HRQL over a relatively long period of time.

Positive treatment results of HLE following HNC have been seen in other research studies [13–15], but there are some differences in study design compared to the present study. Previous studies have been comprised of mixed patient populations, both within the control and intervention groups (cardiovascular accident, CVA, and HNC) and smaller group sizes (11 participants in each study) [13, 14]. A similarity is that treatment was offered some time after completion of oncological treatment. It is possible that participants with impairment due to CVA had better benefit of the exercise than HNC participants since the nature of dysphagic symptoms differ. HNC patients are likely to suffer from side effects of radiotherapy such as neuropathy, edema, and fibrosis. Fibrosis has been noted in some degree as early as 3 months post radiotherapy in a majority of patients and is considered a contributor to dysphagia among HNC patients [29]. Several studies have evaluated the effectiveness of HLE as one out of several exercises within preventive exercise protocols [30–34]. Evidence cautiously points towards better maintenance of the swallowing function by preventative swallowing exercises [10]. Ohba et al. is to our knowledge the only study that has evaluated the effect of the HLE as a singular preventative exercise [15]. The authors found better maintained swallowing function and physiology among participants who performed the HLE as a preventive measure compared to a historical control group who started practicing the Mendelsohn maneuver at onset of dysphagia. Ohba et al. concluded that the HLE as a preventive exercise helps to preserve the swallowing function. Consequently, timing of the HLE intervention might in part explain the lack of effect observed in the present study and the studies of objective swallowing function recently presented by the research group. Although, one thing to bear in mind concerning the study by Ohba et al. is that the HLE group contains participants that might not develop dysphagia, while all participants in the historical control already had established dysphagia. There is also a weakness within the HLE protocol itself. The HLE protocol does not progressively increase the exercise load, an important aspect in building muscular strength [35]. One could increase the resistance by longer sustained head lifts and/or increasing repetitions but this would make the protocol more extensive, risking decreased exercise compliance.

If the HLE had been effective in improving swallowing function, the intervention group should have reported better results than the control group in at least some of the included domains of the HRQL instruments. In other studies evaluating preventive swallowing exercises for HNC patients, MDADI has, on the one hand, detected improvements by intervention in all domains and in total score [31, 36], meanwhile, in another study, demonstrated improvement for the control group in the functional domain [32]. Previous research on how dysphagia relates to general HRQL have shown negative influence of dysphagia in all domains of the EORTC QLQ-C30 and for swallowing, social eating, sticky saliva, and social contact on the H&N35 [24, 37].

The intervention group did report their function as improved in swallowing on QLQ-H&N35 and for some of the domains of the MDADI after 8 weeks of HLE. This indicates that the participants felt more confident in spite of their swallowing difficulties, and that the difficulties posed less trouble in their daily lives. After 12 months, this positive trend had subsided. As previous studies within the research group found no improvement of the HLE in objectively measured swallowing function [16, 17], these improvements might be an effect of the closer contact with the study SLPs during the first 8 weeks of study participation. At the 12-month follow-up, the control group reported statistically significant improvements compared to baseline within a majority of the MDADI domains, while the intervention group reported none. These results might reflect higher expectations for functional improvement within the intervention group due to the effort put into training [38]. Foremost, the results highlight the importance of randomized control trials and subsequent follow-ups as the improvement in dysphagia-specific HRQL at the 8-week follow-up among participants practicing the HLE otherwise could risk being overstated.

It is essential to measure adherence to exercise when evaluating effect of an intervention. Although the HLE is a strenuous exercise to perform, and high intensity is a factor known to influence compliance negatively, adherence in the present study was high throughout the 12 months [12, 39]. In previous studies of swallowing exercises, compliance has been low, making it difficult to draw conclusions [38]. Several factors important to compliance to dysphagia intervention were used in the present study, such as participants receiving instructions and supervision by the study SLP, prescription of exercise dose, and use of a diary for self-monitoring [38].

We know from previous studies done on the present cohort that 33% of patients presented with PAS of ≥ 5 on FEES [17], i.e., penetration to true vocal folds or aspiration, which is in line with earlier research on prevalence of penetration–aspiration events in HNC populations [25, 40]. Furthermore, when comparing total score on the MDADI to results from other reported HNC cohorts primarily treated with radiotherapy at corresponding timepoints, the present study indicated slightly worse results [41, 42]. Therefore, it does not seem like the cohort in the present study was better off compared to other HNC patients in general, consequently difference between the groups should have been possible to detect. Results on EORTC QLQ-C30, on the other hand, were corresponding to those of healthy reference groups throughout the 12 months, indicating that the general HRQL within the cohort was fairly good, which restricted the possibility for change [43, 44]. This was not true for HRQL as measured by the QLQ-H&N35, where values in general were below those of a healthy reference group [45, 46].

The study participants in the present study knew which group they were allocated to, which may be considered a limitation. The study is strengthened by the fact that the adherence to exercise was high throughout the study. Furthermore, comparison of sociodemographic data as well as nutritional and swallowing variables revealed no statistically significant differences between the intervention and control group at either study start or at 12 months.

## Conclusions

The HLE is an intense exercise and quite an undertaking on the part of the patient. No convincing results of improvement have been found on patients’ perception of general and dysphagia-specific HRQL over 12 months in the present study. Relying on results from the present and from previous work within the research group where swallowing function and physiology after 8 weeks of intervention was evaluated by objective measurements, the HLE alone does not seem to be an intervention that significantly improves swallowing function or HRQL in patients with established dysphagia after radiotherapy.
